# Lessons From the Pandemic for Hand Surgery in Wales

**DOI:** 10.7759/cureus.56577

**Published:** 2024-03-20

**Authors:** Owen J Lawrence, Vasudev Shanbhag

**Affiliations:** 1 Orthopaedics and Trauma, Nevill Hall Hospital, Abergavenny, GBR

**Keywords:** hand trauma management, elective hand surgery, quality improvement research, walant principles, covid-19 pandemic, qualitative study

## Abstract

Aims

In March 2020 the World Health Organisation (WHO) declared the COVID-19 virus a global pandemic. The United Kingdom's National Health Service (NHS) was placed under unprecedented pressure and hospitals were forced to adapt their working practices to continue offering world-leading healthcare.

This project aims to highlight the lessons learnt within hand surgical departments throughout Wales. Using this knowledge, we can consider how these lessons can be implemented in both emergency and elective hand practice.

Methods

A qualitative questionnaire was distributed to hand consultants working across Health Boards within Wales during the pandemic. The questionnaire encompasses the impact of the pandemic on usual practices and what local departmental changes have been implemented in response to patient needs.

Results

Across the Welsh Health Boards, we received 12 of 19 consultant responses achieving a 63% response rate and captured data from five of seven (71%) major health boards. The questionnaire revealed that 100% of respondents changed their routine management of elective cases whilst 83% changed their management of hand trauma. 50% reported the need to issue updated management guidelines to junior doctors. The major highlighted lessons were the importance of a dedicated hand fracture clinic, coupled with a ring-fenced day-surgical unit (offering regional anaesthetic support) to manage trauma and elective patients independently from general trauma.

Conclusion

This qualitative research demonstrates that the pandemic drove the restructuring of many hand departments enabling us to find new, efficient ways of working. We must take these lessons forward to tackle the ever-growing waiting list, increased patient expectations and increasingly complex workloads.

## Introduction

In March 2020 the World Health Organisation (WHO) declared the COVID-19 virus a global pandemic and since this time the world has become a different place. As of February 2023, there have been a reported 218,405 deaths due to COVID-19 in the United Kingdom, with 11,445 of those deaths being in Wales [1]. The United Kingdom's National Health Service (NHS) was placed under unprecedented pressure and hospitals were forced to adapt their working practices to continue offering world-leading healthcare whilst protecting both staff and patient safety. 

In January 2021 the British Orthopaedic Association (BOA), in conjunction with the British Society for Surgery of the Hand (BSSH) released guidelines on the management of hand trauma during the COVID-19 pandemic [2]. Within this guideline it was advised that all hand trauma be treated non-operatively where safe and reasonable, that all surgery should be performed under WALANT (Wide Awake, Local Anaesthetic, No Tourniquet) principles and face-to-face appointments should be minimised with most being carried out via video/telephone. Additionally, the BSSH released guidelines on the management of elective hand conditions during the COVID-19 pandemic which stated that the care of non-emergency hand and wrist patients should mainly be suspended during the pandemic [3].

Though there was a significant fall in musculoskeletal trauma, in part due to the ‘Protect the NHS’ campaign, the demand for hand trauma care remained high during the pandemic [4]. Each department rose to the challenge in their own unique way to deliver effective care whilst maintaining all precautions against the COVID-19 virus. An international survey of 47 hand surgeons revealed that only 11% of hand surgeons did not change their practice whilst 83% made significant changes to their operational pathways [5].

This project aims to highlight the key challenges faced within hand surgical departments throughout Wales during the COVID-19 pandemic. Using this information, we can identify key lessons that can be implemented in both emergency and elective hand practice to improve patient care.

## Materials and methods

Study design

In this study, a qualitative study was conducted to collect the opinions of experts on the effects of the COVID-19 pandemic on hand surgery in Wales.

Study participants

As the study aimed to collect expert opinion, the research population was confined to trauma and orthopaedic surgeons with a specialist interest in hands, working within Wales at the time of the pandemic. Through use of the BSSH website and confirmation via the senior author, 19 consultant hand surgeons were identified across Wales, reflecting practice in all major Health Boards across Wales.

Data collection tool and technique

The questionnaire encompasses both elective and trauma practice, with 10 focused questions directed at trauma and nine focused questions directed at elective practice. It was created by the paper's authors and has not been externally validated. The topics covered included clinic utilisation and patient management, theatre utilisation and higher managerial structural changes. The questionnaire consisted of both open and closed questions, with some limited free-text options [6]. Through the use of open questions and free text, we aimed to identify key themes emerging from across the health boards [7].

The questionnaire was distributed via email to consultant hand surgeons working within Wales during the COVID-19 pandemic. The questionnaire was conducted between 01/06/2022 and 31/07/2022. These Health Boards included Aneurin Bevan University Health Board, Betsi Cadwaladr University Health Board, Cardiff and Vale University Health Board, Cwm Taf Morgannwg University Health Board, Hywel Dda University Health Board, Powys Teaching Health Board and Swansea Bay University Health Board.

Across the response time frame, all questionnaires were followed by two reminder emails and a final data collection date to maximise returns. All responses were confidential, with the name of the health board used as the only identifier. Questionnaires were transcribed to Microsoft Excel (version 2401) and this software was utilised for all subsequent data analysis. Free text options were assessed for recurring keywords and themes across the health boards.

Ethical approval was not required for this research as determined by the NHS Research Ethics Commitees (RECs) online evaluation tool.

## Results

**Figure 1:**
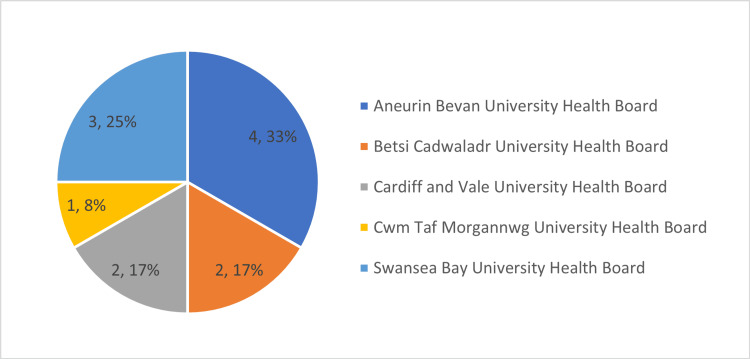
Chart Depicting the Number of Responses to the Questionnaire From the Respective Health Board Within Wales

Across the Welsh Health Boards, 19 consultants who have a specialist interest in hand surgery were invited to take part. We received 12 responses with a 63% response rate and this data captured five of the seven (71%) major health boards within Wales (Figure 1).

Trauma practice

Within the questionnaire, trauma and elective practice were assessed individually. In relation to trauma practice, 83% (10) of responders had to change their usual management of hand trauma cases during the pandemic, with 50% (6) issuing updated treatment guidelines to junior doctors working within their department.

**Figure 2:**
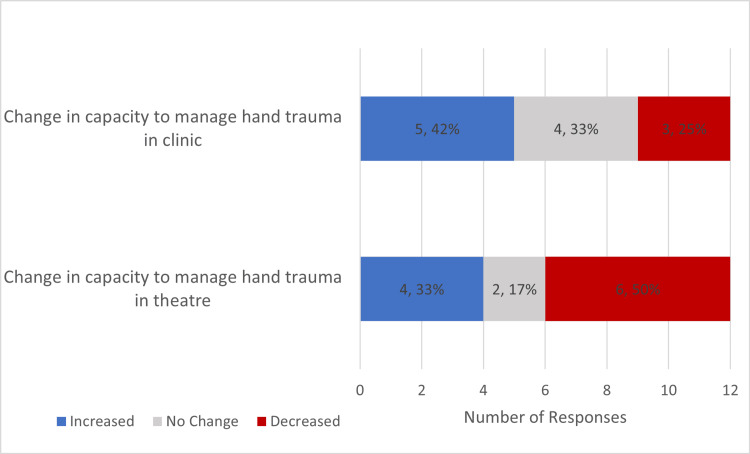
Chart Highlighting Change in Practice Seen in Trauma Setting due to the COVID-19 Pandemic

Regarding capacity, 25% (3) of responders reported decreased capacity to see patients in hand trauma clinics while 42% (5) reported an increase in capacity. Similarly, 50% (6) of responders reported decreased capacity while 33% (4) reported increased capacity in managing hand trauma in theatre (Figure 2).

Whilst reviewing patients in the clinic, 67% (8) of responders stated that patients were discharged from the clinic earlier on their treatment pathway than they otherwise would have been, though two of these responders noted that this was primarily in the early stages of the COVID-19 pandemic.

**Figure 3:**
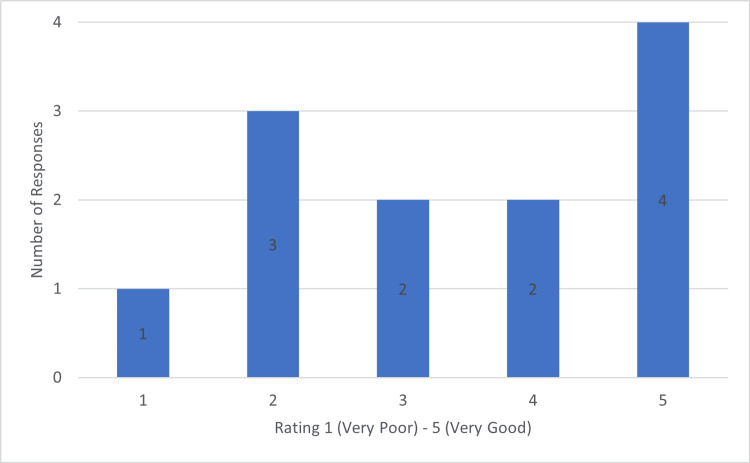
Chart Demonstrating Rating of Postoperative Hand Therapy in Trauma Patients

In theatre, 50% (6) of responders noted that they saw an increase in the use of WALANT (Wide-awake, Local Anaesthesia, No Tourniquet) techniques, whilst 50% (6) noted there was no change as compared to pre-COVID-19. Postoperatively the responders were asked to rate the adequacy of hand therapy available from 1 (very poor) to 5 (very good). The results of which are displayed in Figure 3.

**Figure 4:**
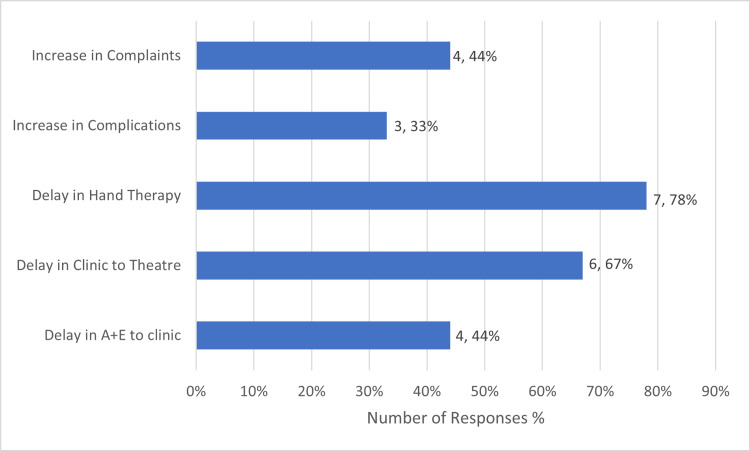
Summary of Negative Effects Noted in Managing Trauma Patients During the COVID-19 Pandemic A+E: Accident and Emergency

Overall, when asked whether they had seen largely positive or negative effects because of management decisions taken during the pandemic, 42% (5) responded positive, 25% (3) no change, 16.7% (2) negative, and 16.7% (2) both positive and negative. The main negative effects noted in trauma practice are summarised in Figure 4.

A final open question covering lessons learnt during the pandemic in trauma practice returned several responses. Key themes emerged which included a move to more conservative management of hand trauma, while also highlighting the importance of separate hand clinics and ring-fenced hand trauma lists to improve. The full transcript of comments can be reviewed in Table 1.

**Table 1:** Key Lessons Learnt in Managing Hand Trauma During the COVID-19 Pandemic A&E: Accident and Emergency

1	Reduced outpatient and theatre provision has led to delays in treatment. Longer wait times following referral has led to sub-optimal outcomes.
2	Post operative hand therapy provision has been reduced to unacceptable levels.
3	Co-locating elective and trauma hands in single unit.
4	Need for the patient to be seen promptly in hand trauma clinic after initial assessment in A&E.
5	Prompt treatment reduces complications.
6	An efficient day surgery unit would have been very helpful.
7	Centralisation to dedicated hand fracture clinic and day case with regional support trauma theatre resulted in increased through put and efficiency and better patient outcomes.
8	More lean and virtual pathways.
9	To ring fence hand trauma from generic trauma management.
10	A lot more conservative treatment we haven’t seen the tsunami of untreated injuries we expected.
11	Non-operative management has seen a revival due to the pandemic.
12	Most hand fractures can be managed non-operatively.

Elective practice

**Figure 5:**
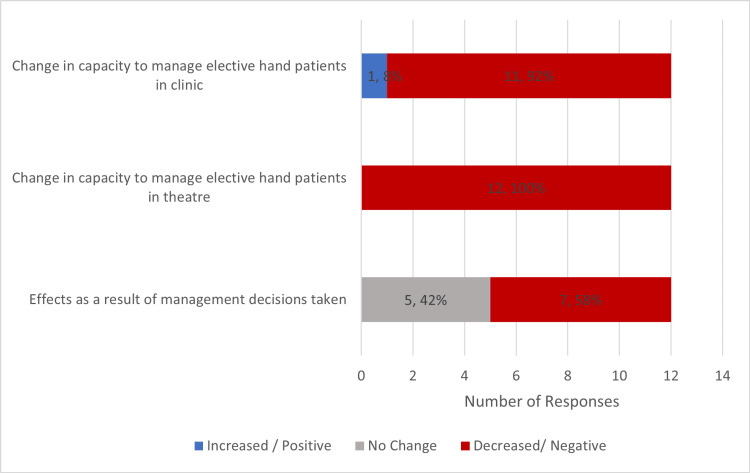
Chart Highlighting Change in Practice Seen in Elective Setting due to COVID-19 Pandemic

In relation to elective practice, 100% (12) of responders reported that they were required to change their typical management of elective hand cases. 92% (11) of responders reported decreased clinic capacity and 100% (12) of responders noted that there was a decrease in theatre capacity. In assessing the effects of this 58% (7) of responders felt that there was an overall negative impact because of management decisions taken during the pandemic (Figure 5).

**Figure 6:**
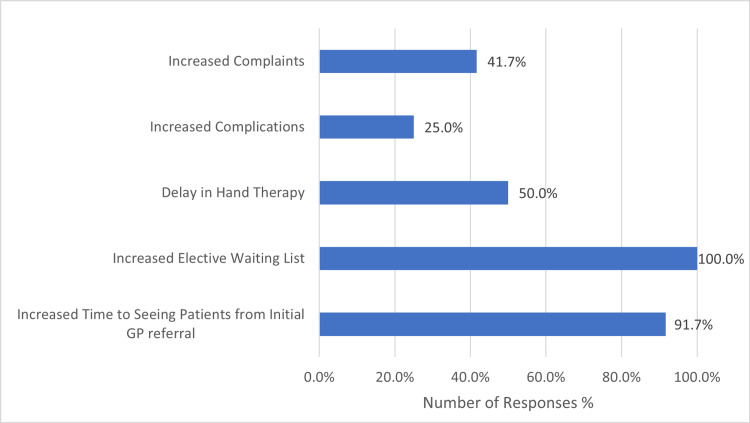
Chart Demonstrating Summary of Negative Effects seen in Elective Practice due to COVID-19 Pandemic GP: General Practitioner

Looking further into the impact, all consultants noted at least one negative impact, with 92% (11) seeing increased time to assessment from original General Practitioner (GP) referral, and 100% (12) of responders seeing increased elective waiting lists. 50% (6) of consultants saw a delay in appointments with hand therapy and 42% (5) of responders found that they had seen an increased number of complaints being received (Figure 6).

**Figure 7:**
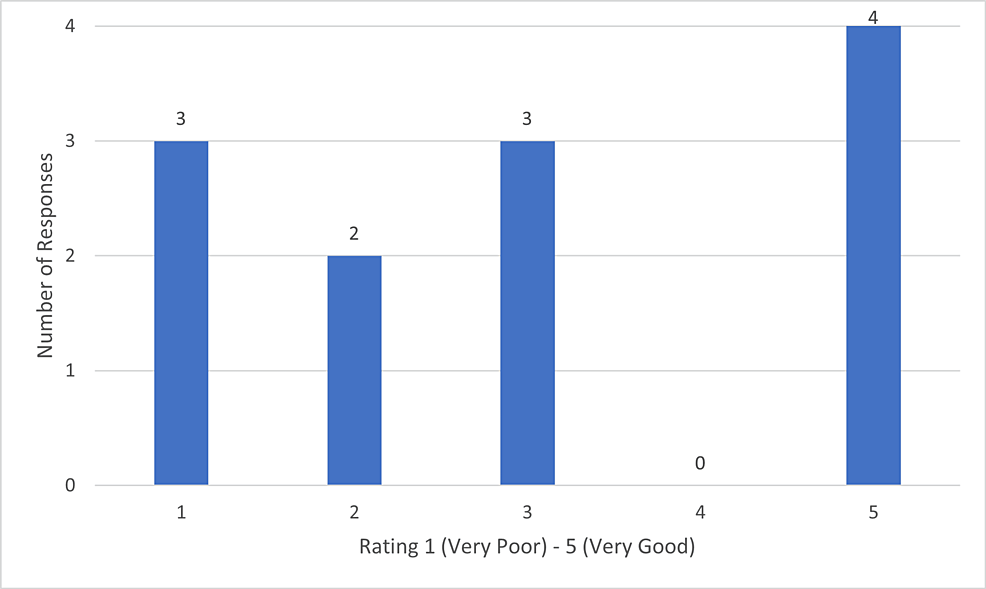
Chart Demonstrating Rating of Postpperative Hand Therapy in Elective Hand Patients

In theatre, 83.3% (10) of responders noted that they saw no change in the use of WALANT techniques. Postoperatively the responders were asked to rate the adequacy of hand therapy available from 1 (very poor) to 5 (very good). The results of which are displayed in Figure 7.

A final open question covering lessons learnt during the pandemic in elective practice returned several responses, with many highlighting their feeling that day case elective hand surgery should not have been stopped during the COVID-19 pandemic. Some responders noted that there was still an ongoing reduction in capacity and that there was a strong need for ring-fenced day surgery hubs, with greater capacity within the Hand Therapy service. The full transcript of comments can be reviewed in Table 2.

**Table 2:** Key Lessons Learnt in the Management of Elective Hand Cases During the COVID-19 Pandemic

1	Increased waiting times for referral and treatment have had a deleterious outcome for elective hand patients. disease progression has been worrisome, with reduced opportunity for successful surgical intervention.
2	None specifically (elective work unable to continue for many months during the worst of the pandemic).
3	A surprising number of patients elected to be discharged off the waiting list rather than consider coming into hospital. It made me wonder how many of them really needed treatment in the first place.
4	We should have kept elective services running as best we could, instead of stopping completely.
5	We should not have stopped elective operating as lessons from trauma management in a day case fashion showed no increase in COVID patient who were appropriately screened pre-op.
6	We still have significantly reduced capacity for elective hand surgery.
7	Need to have more resources specially from Hand Therapy perspective Will need more ring-fenced Day Surgery Hubs.
8	The tunnel is getting longer with no light at the end visible.
9	Massive waiting lists due to shortage of elective capacity.

## Discussion

We performed qualitative research to assess the effects of COVID-19 on hand surgery practices throughout Wales. Through our research, we have achieved a snapshot that has included 71% of major health boards in Wales to give us a true reflection of practice.

Trauma practice

During the COVID-19 pandemic, NHS services were being overwhelmed by the additional patient burden the virus created. To reduce other admission types, the UK government launched multiple advertisement campaigns with the slogan “Stay Home, Protect the NHS, Save Lives”[8]. Though trauma admissions did reduce with some centres reporting a 56% reduction, hand trauma remained common, being the third most common musculoskeletal area injured after hips and ankles [9].

Within Wales, we can see that the hand departments took on the initiative from the UK government to reduce admissions. 83% (10) of consultants surveyed changed their usual management of hand trauma and 50% (6) issued updated treatment guidelines to junior doctors. This was within keeping with the BOA/BSSH guidelines released in 2021 which aimed to manage a significant proportion of hand trauma in Accident and Emergency with discharge home [2]. From our data, there was a mixed picture of theatre availability to manage these patients, largely due to local factors within each health board. Venkatesan et al. [10] highlighted the reconfiguration strategies that were being implemented across the UK. Within this study responders commented on the need to ‘ring fence’ hand trauma theatre lists, reflecting the logistical difficulty of prioritising hand trauma on sometimes overwhelmed general trauma theatre lists. Athar et al. [11] studied the efficiency of day case trauma list services and found them to be a safe option that is cost-effective and yields high patient satisfaction rates. Through the questionnaire, it appears that this is highly desirable within hand services and is gradually being implemented throughout Wales.

The findings from theatre provision are largely reflected in the trauma clinic survey responses. 41% (5) of responders reported an increased capacity to see hand trauma patients in the clinic whilst 25% (3) reported a decreased capacity. Patients were discharged earlier in keeping with BSSH guidelines, but it was noted that there was an increased rate of complaints and complications. Specific responders commented on the need for a centralised and dedicated hand fracture clinic.

Combining the questionnaire responses for managing hand trauma in clinics and theatres, it appears that the COVID-19 pandemic has created or accelerated a want to streamline hand services within Wales. Though in many hospitals this has historically been in place, the questionnaire responses show a clear wish to provide hand trauma services through a dedicated hand fracture clinic and ‘ring-fenced’ hand trauma theatre lists.

Elective practice

In the early phase of the COVID-19 pandemic, the risk to patients undergoing elective procedures was not fully understood. As such guidelines published by the BSSH stated that the care of non-emergency hand and wrist patients should mainly be suspended [3]. This research demonstrates Wales's application of these guidelines with 100% (12) of responders reporting reduced capacity in theatre and 92% (11) reporting reduced capacity in clinics to manage elective hand pathology. As further data was explored the BOA, in partnership with BSSH and BOFAS (British Orthopaedic Foot and Ankle Society), produced updated guidelines on the resumption of local anaesthetic musculoskeletal procedures [12]. These expressed that in controlled pathways, to protect both patient and staff, local anaesthetic without sedation, including Wide-Awake Local Anaesthesia No Tourniquet (WALANT) techniques could be implemented.

WALANT surgery is an attractive option for hand surgeons as procedures can be performed safely and efficiently with reduced patient anxiety, reduced post-operative analgesia requirements and reduced cost as compared to the same surgery performed under General Anaesthesia [13]. Within our study, 83% (10) of respondents reported no change in the use of WALANT principles whilst 17% (2) reported an increase. Historically, there has been slow adoption of WALANT procedures due to multiple areas of resistance; anaesthetic and perioperative staff feeling “cut out” from these procedures, questions related to patient comfort and health boards being caught in a historical mindset.

The COVID-19 pandemic has rejuvenated the drive towards WALANT principles in hand surgery. Kurtzman et al. [14] completed a case series of 72 patients who underwent WALANT procedures in New York during the COVID pandemic, whilst Sutcliff et al. [15] reviewed 37 patients who underwent similar procedures performed in a UK hospital. In both cases, no complications were reported, and patient satisfaction scores were stated as high. These studies re-iterate that hand surgery can be performed effectively using WALANT principles and is an excellent tool to utilise in areas where access to a main theatre complex or ventilatory support is limited.

Study limitations

In this study, our questionnaire had a response rate of 63%, and this is one of the study's largest limitations. As this research focused on hand consultants within Wales our overall target demographic was limited to 19 consultants. Despite this we did gain data on five out of the seven major health boards, missing data on Hywel Dda University Health Board and Powys Teaching Health Board. Given the response rate it is possible some themes were missed, though within the responses we had, opinions were largely in parallel.

If a qualitative research project such as this were to be completed within Wales again, the inclusion of Speciality Registrars with a special interest in hands may increase both the response rate and spread across health boards.

## Conclusions

This qualitative research demonstrates that the pandemic drove the restructuring of many hand departments enabling us to find new, efficient ways of working. In the post-pandemic era, we will be faced with ongoing staff shortages, an ever-growing waiting list and the emergence of ‘the lost’ patients. It is extremely important to highlight the beneficial changes to practice that the pandemic has forced upon us. Within Wales, this questionnaire has highlighted the importance of streamlining services with dedicated hand fracture clinics, dedicated hand trauma theatres, ‘ring-fenced’ hand beds and the incorporation of WALANT principles. We must take these lessons forward to tackle the ever-growing waiting list, increased patient expectations and increasingly complex workloads.
